# Infantile fever-triggered acute liver failure caused by novel neuroblastoma amplified sequence mutations: a case report

**DOI:** 10.1186/s12876-020-01451-4

**Published:** 2020-09-21

**Authors:** Weiran Li, Yu Zhu, Qin Guo, Chaomin Wan

**Affiliations:** 1 Department of Paediatrics, West China Second University Hospital, Sichuan University, No 20, 3rd section of Renmin South Road, Chengdu, 610041 PR China; 2 Key Laboratory of Birth Defects and Related Diseases of Women and Children (Sichuan University), Ministry of Education, Chengdu, PR China

**Keywords:** NBAS, Acute liver failure, Infantile liver failure syndrome type 2, Whole exome sequencing

## Abstract

**Background:**

Infantile liver failure syndrome-2 (ILFS2) is caused by neuroblastoma amplified sequence (NBAS) mutation. The disease is characterized by recurrent episodes of acute liver failure (ALF) or by liver crisis triggered by recurrent episodes of fever and complete recovery.

**Case presentation:**

Here, we describe the case of a Chinese girl with typical clinical manifestation of ILFS2 without exhibition of extrahepatic involvement. The patient harbored novel compound heterozygous mutations in the NBAS region (c.3386C > T (p.Ser1129Phe), c.1A > C (p.Met1Leu) and c.875G > A (p.Gly292Glu)), mutations which have not been previously reported. After administration of antipyretics and intravenous glucose and electrolyte administration, the patient recovered fully.

**Conclusion:**

Through the present study, we recommend that ILFS2 should be taken into consideration during the differential diagnosis of children with recurrent, fever-triggered ALF. While the definitive diagnosis of ILFS2 remains dependent on genetic sequencing and discovery of NBAS, early antipyretic treatment is recommended to prevent liver crisis.

## Background

Acute liver failure (ALF) is a rare condition in children, however, it is associated with a higher risk of mortality. Although the etiology of ALF in about 50% of cases remains unexplained [1], hereditary metabolic disorders comprise of a large number of ALF in pediatric populations [2, 3]. Recently, biallelic mutations in the neuroblastoma amplified sequence (NBAS) were reported as a novel cause of infantile liver failure syndrome-2 (ILFS2). ILFS2 is an autosomal recessive genetic disease characterized by recurrent episodes of ALF or liver crisis triggered by fever. Previously, the mutations within the NBAS were reported to be the cause of short stature, optic atrophy and Pelger-Huët anomaly of granulocytes (SOPH) syndrome in an isolated Russian yakut population [4]. Increasing research has reported that the phenotype spectrum of NBAS mutations ranges from isolated ILFS2 to a multi-systemic disease including short stature, skeletal dysplasia and optic atrophy [5–8]. Early administration of antipyretic and support therapy can effectively ameliorate the course of ILFS2 due to NBAS mutations, improving the prognosis [8, 9]. Thus, it is important to confirm the diagnosis of ILFS2 at the earliest stage possible. Here, we report a case of isolated ILFS2 caused by novel NBAS mutation in a Chinese girl aged 3 years and 8 months. This report also reviews NBAS-related cases in the medical literature.

## Case presentation

A 3 year and 8 month old Chinese girl born at term to healthy, non-consanguineous parents by normal vaginal delivery had no family history of liver disease. At the age of 2 years and 11 months, the patient presented recurrent fever with cough, vomiting, decreased activity, and lethargy agitated for 3 weeks upon admission to our hospital. She had severe jaundice without hepatomegaly, however the rest of the physical examinations (growth and development, skeletal system, nervous system and motor ability, integument and facial profile) were normal. Laboratory evaluations indicated extreme damage to liver functions and severe coagulopathy (Table 1). Serum creatinine (Cre) and blood urea nitrogen (BUN) were normal. Creatine kinase (CK) and cardiac troponin I (cTnI) were mildly elevated at 149 U/L (normal range: 30–135) and 0.055μg/L (normal range: 0–0.034) respectively, suggesting myocardial injury. The white blood cell (WBC) count was significantly increased (15.8*10^9^/L, normal range:3.6–13) and neutrophils accounted for 81% (12.77*10^9^/L, normal range:0.72–4.80). C-reactive protein (CRP) was also elevated (39 mg/L, normal range:0–8). The patient was diagnosed with ALF and managed with antipyretics, plasmapheresis, hepatoprotectives (e.g., Polyene Phosphatidyl Choline and Ademetionine Butanedisulfonate) and intravenous infusion of glucose and potassium chloride. Following treatment, the liver function of the patient was restored completely.

**Table 1:** Clinical features and laboratory results of the presented patient

Clinic features & laboratory results	Result		
	Age of 2 year and 11 months	Age of 3 year and 7 months	Age of 3 years and 8 months
Symptom	Recurrent fever	Recurrent fever with cough	Fever with vomit
Body sign	Severe Jaundice	Mild Jaundice, Hepatomegaly	Hepatomegaly, A few bleeding spots in her chest, Herpes in right soft palate, An ulcer (3*3 mm) in right isthmus of fauces
Max temperature (°C)	>38.5	39.0	39.3
ALT(U/L) (Normal range:9–52)	9787	6378	4096
AST (U/L) (Normal range:14–36)	12,489	9924	4857
TBIL (umol/L) (Normal range:3–22)	44.3	22.4	28.4
DBIL (umol/L) (Normal range:0–5)	24.10	7.30	16.9
IBIL (umol/L) (Normal range:0–19)	20.20	15.10	11.50
Albumin (g/L) (Normal range:35–50)	41.9	44.1	36.8
γ-GT (U/L) (Normal range:12–43)	26	48	42
LDH (U/L) (Normal range:313–618)	21,786	22,854	6492
PT (s) (Normal range:8.7–14.7)	63.9	15.6	25.9
INR (Normal range:0.8–1.5)	5.50	1.48	2.47
APTT (s) (Normal range:17.5–37.5)	45.0	25.1	25.5
FIB (mg/dL) (Normal range:200–400)	221	327	310
TT (s) (Normal range:14–21)	24.3	20.2	19.1

*NA* Not available, *ALT* Alanine transaminase, *AST* Aspartate aminotransferase, *TBIL* Total bilirubin, *DBIL* Direct Bilirubin, *IBIL* Indirect Bilirubin, *γ-GT* γ-glutamyl transpeptidase, *LDH* Lactate dehydrogenase, *PT* Prothrombin time, *INR* International normalized ratio, *APTT* Activated partial thromboplastin time, *FIB* Fibrinogen, *TT* Thrombin time

Eight months later, at the age of 3 years and 7 months, the patient was readmitted during a second episode of liver crisis following an upper respiratory tract infection with fever (up to 39 °C) and cough. Physical examinations revealed mild jaundice with an enlarged liver (palpable 2.5 cm below the costal margin). Laboratory data indicated a recurrence of significant damage of liver function and severe coagulopathy (Table 1). The value of CK, cTnI, Cre and BUN were normal during this episode and the trends of WBC and CRP were similar to those of the first episode. Again, after treatment including antipyretics, antibiotics, hepatoprotectives and intravenous glucose and potassium chloride, the patient fully recovered.

At the age of 3 years and 8 months, the patient was once again re-admitted with ALF after a 12 h persistence of fever (maximum temperature: 39.3 °C) and vomiting. Physical examination revealed hepatomegaly (palpable 1.5 cm below the costal margin), a few bleeding spots in her chest, and herpes in her right soft palate along with an ulcer (3*3 mm) in the right isthmus of fauces. No jaundice was observed. The clinical features and elevation of liver enzymes with coagulopathy were similar to previous episodes (Table 1). Coxsackievirus A16 nucleic acid detection was positive. Other laboratory results presented similar changes to those observed during the second episode. Complete recovery of the patient was achieved with a similar treatment plan to the previous admission.

During the three admissions, metabolic evaluations indicated hyperammonemia (40.0 umol/L; Normal range:9–30) and hypoglycemia (3.77 mmol/L; Normal range:4.1–5.9). Lactate, ceruloplasmin, alpha-fetoprotein and pyruvate values were all normal. However, there was a remarkable elevation of β-hydroxybutyrate (1.81 mmol/L; Normal range:0–0.27). Serum amino acid test presented elevations of pipecolic acid (551.318 μM; normal range: 100–438) and the ratio of leucine to alanine (1.359; normal range:0.06–1.00). The values of acyl carnitines were normal in the plasma. Metabolic biomarkers in the urine showed that the value of ketone bodies, methionine, adenine and adenosine were all increased. The immunological indicators including complement C3 and C4 levels, serum immunoglobulin levels and the count of T lymphocyte, B lymphocyte, natural killer cells, and helper T cells were all unremarkable. Further, a panel of serology tests for infectious agents including HIV virus, treponema pallidum, hepatitis B virus, hepatitis C virus, Epstein-Barr virus, toxoplasma, rubella virus and herpes simplex virus were all negative. No Pelger–Huët anomaly of granulocytes was found in either of multiple blood smears. A percutaneous liver biopsy was undertaken at the third admission. This showed nonspecific, mild acute hepatitis with hydropic degeneration of hepatic cells and small foci of lobular necrosis. There were a small number of lymphocytes, monocytes and plasma cells infiltrating in the portal area. Foot and Masson stain showed fibroplasia and the enlargement of some portal areas. Berlin blue, rhodanine, rubeanic acid, Periodic Acid-Schiff and diastase peniodic acid-Sehiff stain were normal. In order to identify the cause for recurrent ALF, whole exome sequencing was performed in the patient and her parents. Three rare heterozygous mutations in the NBAS gene were detected: *c.3386C > T (p.Ser1129Phe)*, *c.1A > C (p.Met1Leu)* and *c.875G > A (p.Gly292Glu)*, the latter of which have not been previously reported. Parental genotyping showed that the *c.3386C > T (p.Ser1129Phe)* and *c.875G > A (p.Gly292Glu)* mutation occurred in a heterozygous state in the mother and the *c.1A > C (p.Met1Leu)* mutation in a heterozygous state in the father, presenting compound heterozygosity of the NBAS variants in the girl. According to the guidelines of The American College of Medical Genetics Genomics and the Association for Molecular Pathology (ACMG), the pathogenicity of the *c.1A > C (p.Met1Leu)* mutation was greater than 90%. The pathogenicity of the other two mutations, however, was uncertain. Prediction of protein biological function was carried out with software including SIFT, MutationTaster and GERP++, which indicated that two of the mutations (*c.3386C > T (p.Ser1129Phe)* and *c.1A > C (p.Met1Leu))* were pathogenic while the *c.875G > A (p.Gly292Glu)* mutation was nonpathogenic. Specifically, for the mutation of c.3386C > T (p.Ser1129Phe), the score and prediction of SIFT, MutationTaster and GERP++ were (0.001, damaging), (1, disease causing) and (5.74, conserved) respectively. Regarding the mutation of c.1A > C (p.Met1Leu), the score and prediction of SIFT, MutationTaster and GERP++ were (0, damaging), (1,disease causeing) and (5.2, conserved) respectively. For the mutation of c.875G > A (p.Gly292Glu), these values were (1, tolerated), (1, polymorphism) and (− 3.5, nonconseved) respectively.

## Discussion and conclusions

ILFS2 is a rare condition characterized by episodic liver failure or crisis triggered by recurrent fever. Liver function can be fully recovered after conservative treatment. It was first reported by Haack et al. that ILFS2 is caused by biallelic mutations in the NBAS gene [10]. NBAS mutations have also been identified in SOPH syndrome patients without liver failure [4]. An increasing number of studies have indicated that diseases based on NBAS mutations have a broad phenotypic spectrum, ranging from isolated recurrent ILFS2 to a multi-systemic disease manifesting as short stature, skeletal dysplasia, dysmorphism and optic atrophy with or without liver failure [4–9, 11–18].

A literature search found 56 reported cases of NBAS-associated disease (Table 2). The median onset age of these patients was 0.8 years (interquartile range:0.1–6.7 years) however only 47 patients were considered due to missing data of onset age. The ratio of female to male patients was 17 to 13, however, only 30 patients were analyzed due to incomplete data of sex. The clinical manifestations of the hereditary disease were characterized by two aspects including intrahepatic and extrahepatic symptoms. On the one hand, the phenotype of liver illness is usually triggered by febrile illness and manifested as recurrent ALF or liver crisis, where complete recovery is typical during the interval of episodes. It usually begins with rapid increased value of alanine transaminase and aspartate transaminase, indicating functional liver damage. Mild to severe coagulopathy, jaundice and hepatomegaly are manifested in most cases. Secondary metabolism disorders, such as elevation of lactate and serum ketone bodies, hypoglycemia, and hyperammonemia are sometimes observed in these patients [7, 9]. Moreover, some patients present hepatic encephalopathy or death due to untimely treatment. On the other hand, the main extrahepatic manifestation of NBAS mutations are short stature, skeletal dysplasia, optic atrophy, Pelger-Huët anomaly of granulocytes or immunological abnormalities [7, 13, 17]. Previous researchers have reported that among the 56 reported patients with NBAS-associated disease, 43 patients (76.8%) presented ILFS2 with multi-systemic manifestations, where short stature was the most common extrahepatic manifestations (34/43, 79.1%). Only 13 cases (23.2%) presented isolated ILFS2 (Table 2). The present case reported a 3 years and 8 months old girl with ILFS2 who experienced the first episode at 2 years and 11 months of age and it presented herein details isolated ILFS2 with coagulopathy, jaundice and hepatomegaly, where no extrahepatic abnormalities were observed. We speculated that the clinical manifestations may be associated with the different gene mutation profile of this patient.

**Table 2:** Clinical features of patients with NBAS deficiency reported in previous studies

Patient ID	Sex	Nationality	Genotype	Onset age of ALF or LC (Year)	Age at last visit (Year)	Number of ALF	Number of LC	ALF or LC Fever depended	Skeletal system and musculature	Typical morphological characteristics	Integument (Cutis laxa)	Nervous system	Immune system	Pelger Huët anomaly	Other clinical manifestations
1	F	Germany	c.[558_560del];[686dup] p.[Ile187del];[Ser230Glnfs*4]	1.8	18	2	12	YES	Short stature (− 2.67SD)	NA	NA	Optic atrophy	NO	NO	Hypotelorism
2	F	Germany	c.[2708 T > G];[2708 T > G] p.[Leu903Arg];[Leu903Arg]	0.6	22	7	6	YES	Short stature (−2.08SD)	NA	NA	Epilepsy; Learning disability (IQ 77)	NO	NO	Acute renal failure; Hypotelorism
3	F	Germany	c.[603_605del];[3164 T > C] p.[Leu202del];[Leu1055Pro]	0.8	18	5	8	YES	Short stature (−4.24SD)	NA	NA	NO	NO	NO	Hypotelorism; disease
4	F	Germany	c.[2708 T > G];[2827G > T] p.[Leu903Arg];[Glu943*]	0.7	37	NA	NA	YES	Short stature (−2.25SD)	NA	NA	Coordination deficit Intellectual disability (IQ 50)	NO	NO	Hypotelorism
5	M	Germany	c.[3010C > T];[3164 T > C] p.[Arg1004*];[Leu1055Pro]	0.6	14	10	4	YES	NO	NA	NA	NO	NO	NO	Hypotelorism; Cardiomyopathy
6	M	Great Britain	c.[1533_1545del];[2951 T > G] p.[Ile512Thrfs*4];[Ile984Ser]	1.5	3	5	2	YES	Short stature (−2.91SD)	NA	NA	Motor delay	NO	NA	NO
7	F	Croatia	c.[1042C > T];[2203_3C > G] p.[Pro348Ser];[?]	0.9	9	4	5	YES	NO	NA	NA	NO	NO	NA	Hypotelorism
8	F	Croatia	c.[1042C > T];[2203_3C > G] p.[Pro348Ser];[?]	6.7	11	1	2	YES	Short stature (−2.71SD)	NA	NA	NO	NO	YES	Erythema nodosum; Crohn’s disease
9	M	Germany/ Turkey	c.[1187G > A];[2330C > A] p.[Trp396*];[Pro777His]	1.8	8	1	3	YES	NO	NA	NA	NO	NO	NA	NO
10	F	United States	c.[118-2A > G];[2524G > T] p.[?];[Val842Phe]	1.5	4	3	NA	YES	Short stature (−2.62SD)	NA	NA	NO	NO	NA	Episodes of mild hypoglycemia during acute illness
11	F	United States	c.[686dup];[3164 T > C]p.[Ser230Glnfs*4];[Leu1055Pro]	0.3	18	5	2	YES	Short stature (−2.35SD)	NA	NA	NO	NO	NA	NO
12	M	France	c.[2827G > T];[exons39–40del] p.[Glu943*];[Val2145_Glu2237del]	0.6	1.8	8	0	YES	Short stature (−2.48SD); Skeletal dysplasia; Mild hypotonia;	NA	NA	Optic atrophy; Left hemi-paresis; Motor delay; Epilepsy; Hypotonia;	Hypogammaglobulinemia	Yes	Lymphopenia during crisis; Neuroblastoma
13	M	Germany	c.[1278A > C];[exons49–50del] p.[Cys426Trp]; [Val1528Glyfs*2]	0.8	8	1	3	YES	Short stature (−2.07SD)	NA	NA	NO	Hypogammaglobulinemia	YES	NO
14	F	Germany	c.[173-2A > G];[3363A > G] p.[=];[Ile1121Met]	2	16	12	1	YES	NO	NA	NA	NO	NA	NA	NO
15	F	American	c.[409C > T];[1186 T > A] p.[Arg137Trp];[Trp396Arg]	1.9	5	3	NA	YES	Short stature (−3.5SD); Skeletal dysplasia; Cervical instability; Fractures (1 skull and 1 left femur fracture);	YES	YES	Motor delay	Reduced natural killer cells	YES	NO
16	M	Switzerland	c.[284C > T];[850A > T] p.[Ala95Val];[Lys284Ter]	0.4	12	0	>1	YES	Short stature (−3SD); Skeletal dysplasia; Cervical instability;	YES	YES	Optic atrophy	Hypogammaglobulinemia; Reduced natural killer cells	YES	NO
17	F	NA	c.[2819A > C] p.[His940Pro]	0.3	4	≥1	≥1	YES	Short stature	NO	NO	NO	NO	NA	NO
18	M	Japanese	c.[1018G > C];[2674 G > T] p.[Gly340Arg]; [Val892Phe]	3.3	3.5	2	0	YES	Short stature	NO	NA	NO	NO	YES	Renal failure;
19	F	China	c.[1840delG]; [3596G > A] p.[Ala614Leufs*3]; [Cys1199Tyr]	0.5	0.58	1	2	YES	NO	YES	NO	NO	NA	NA	NO
20	M	China	c.[6751_6754delCTCC]; [3596-G > A] p.[Leu2251Cysfs*5]; [Cys 1199Tyr]	1.2	5.4	≥2	≥3	YES	NO	NA	NA	NO	Reduced natural killer cells	YES	NO
21	M	China	c.[7041_7043delTCT];[3759delC] p.[2347_2348delLLinsL];[Thr1254Hisfs*16]	NA	0.75	NA	≥2	YES	NO	YES	NA	NO	NO	YES	NO
22	F	China	c.[3596G > A];[209 + 1G > A] p.[Cys1199Tyr];[?]	NA	4	NA	≥1	YES	NO	NO	NO	NO	NA	NA	NO
23	M	China	c.[6611_6612insCA];[3596G > A] p.[Met2204Ifs*3];[Cys1199Tyr]	0.5	6 .9	5	3	YES	Short stature (−1.42SD)	NO	NO	NA	NA	NA	NO
24	M	China	c.[3596G > A];[586C > T] p.[Cys1199Tyr];[Gln196X]	0.6	4.7	1	2	YES	Short stature(−1.47SD)	NO	NO	NA	NA	NA	NO
25	M	China	c.[5389 + 1G > T];[2407G > A] p[?];[Glu803Lys]	0.5	2.3	10	1	YES	NO	NO	NO	NA	NA	NA	NO
26	F	NA	c.[2827G > T];[5741G > A] p.[Glu943*];[Arg1914His]	0.7	4.9	NA	≥2	YES	Short stature(−6SD); Skeletal dysplasia; Delayed bone maturation; Hypotonia	YES	NA	Optic atrophy; Motor delay; Speech delay	Reduced B cells; Initially neutropenia; Hypogammaglobulinemia	YES	NO
27	F	Portuguese	c.[680A > C];[1749G > A] p.[His227Pro];[Trp583*.]	2.8	3.9	0	2	YES	Short stature; Poor muscular development; Slightly delayed bone age (18 months at 23 months of age);	YES	NO	NO	NO	NA	NO
28	F	Portuguese	c.[680A > C];[1749G > A] p.[His227Pro];[Trp583*.]	1.5	9	2	>30	YES	Short stature; Low bone mineral density	NO	NO	NO	NO	NA	NO
29	NA	NA	c.[284C > T]; [2802G > A] p.[Ala95Val; [Trp934*]	NA	NA	≥1	NA	NA	Short stature; Cervical instability; Reduced bone mineral density; Muscular hypotrophy	NA	YES	Motor delay	Hypogammaglobulinemia	NO	High pitched voice
30	NA	NA	c.[812 T > C]; [812 T > C] p.[Leu271Pro]; [Leu271Pro]	NA	NA	≥1	NA	NA	Short stature; Muscular hypotonia and hypotrophy	NA	NO	Motor delay	Hypogammaglobulinemia	NO	Protuberant abdomen; High pitched voice
31	NA	NA	c.[1241C > T];[2950delA; p.[Ser414Phe];[Ile984Leufs*8]	0.2	NA	≥1	NA	NA	Short stature; Skeletal dysplasia; Reduced bone mineral density; Pathologic fracture; Muscular hypotonia and hypotrophy	NA	YES	Optic atrophy	Hypogammaglobulinemia	YES	Insulin dependent diabetes mellitus; Chronic lung disease; High pitched voice
32	NA	NA	c.[1241C > T];([6236 + 1_6237–1]_[6432+ 1_6433–1] del); p.[Ser414Phe]; [Glu2080*]	0.7	NA	0	≥1	NA	Short stature; Skeletal dysplasia; Reduced bone mineral density; Muscular hypotonia and hypotrophy	NA	YES	Optic atrophy; Motor delay	NO	YES	Relative macrocephal; Decreased serum IGF1 and IGFBP3; High pitched voice
33	NA	NA	c.[1549C > T];[5041_5048del]; p.[Arg517Cys];[Ser1681Glnfs*37]	0.7	NA	≥1	NA	NA	Reduced bone mineral density; Skeletal dysplasia; Scoliosis; Pathologic fracture; Muscular hypotonia and hypotrophy	NA	NO	NO	Hypogammaglobulinemia	NO	Cataract and dislocation of the lens; Nephrolithiasis; High pitched voice
34	NA	NA	c.[1550G > A]; [6805G > T]; p.[Arg517His]; [Glu2269*]	3.0	NA	≥1	NA	NA	NO	NA	NO	NO	NO	NO	NO
35	NA	NA	c.[1550G > A]; [6805G > T]; p.[Arg517His]; [Glu2269*]	NA	NA	≥1	NA	NA	NO	NO	NO	NO	NO	NO	Preemptive family testing
36	NA	NA	c.[2191A > C]; [2191A > C]; p.[Thr731Pro]; [Thr731Pro]	1	NA	≥1	NA	NA	Short stature	NO	NO	NO	NO	NO	Liver transplantation
37	NA	NA	c.[2330C > A]; [2330C > A]; p.[Pro777His]; [Pro777His]	2.7	NA	≥1	NA	NA	NO	NO	NO	NO	NO	NO	Elevated 3-methylglutaconic in urine; Vitamin D deficiency
38	NA	NA	c.[2330C > A]; [2330C > A]; p.[Pro777His]; [Pro777His]	3.2	NA	≥1	NA	NA	NO	NO	NO	NO	NO	NO	Vitamin D deficiency
39	NA	NA	c.[2809C > G]; ([5138 + 1_5139–1]_7116del) p.[Pro937Ala]; [?]	0.7	NA	≥1	NA	NA	Short stature	NO	NO	NO	NO	NO	NO
40	NA	NA	c.[2819A > C];[2819A > C]; p.[His940Pro];[His940Pro]	0.2	NA	≥1	NA	NA	NO	NO	NO	NO	Reduced NK cells	NO	NO
41	NA	NA	c.[2951 T > G]; [2827G > T]; p.(Ile984Ser); (Glu943*)	2.6	NA	≥1	NA	NA	Short stature; Skeletal dysplasia; Delayed skeletal maturation	NO	NO	NO	NO	YES	Precocious puberty; High pitched voice
42	NA	NA	c.[3164 T > C]; ([5027 + 1_5028–1] _[5724 + 1_5725–1] del); p.[Leu1055Pro]; [Glu1676Aspfs*10]	1.3	NA	≥1	NA	NA	NO	NO	NO	NO	Hypogammaglobulinemia	NO	NO
43	NA	NA	c.[3363A > G]; [513 + 2 T > C]; p.[Ile1121Met]; [?]	1.0	NA	0	≥1	NA	Short stature	NO	NO	NO	Hypogammaglobulinemia	NO	NO
44	NA	NA	c.[3386C > T]; [3386C > T]; p.[Ser1129Phe]; [Ser1129Phe]	2.3	NA	≥1	NA	NA	NO	NO	NO	NO	NO	NO	Elevated 3-methylglutaconic and 3-methylglutaric in urine
45	NA	NA	c.[3386C > T]; [3386C > T]; p.[Ser1129Phe]; [Ser1129Phe]	1.8	NA	≥1	NA	NA	NO	NO	NO	NO	NO	NO	Elevated 3- methylglutaconic and 3-methylglutaric in urine
46	NA	NA	c.[3534C > A];[1342-6A > G]; p.[Ser1178Arg];[?]	0.8	NA	≥1	NA	NA	NO	NO	NO	NO	Hypogammaglobulinemia Reduced NK cells	NO	During crisis: seizures, cholecystitis, truncal exanthema, hypophosphatemia, anemia, lymphopenia, decrease of neutrophils
47	NA	NA	c.[3602A > C]; [3602A > C]; p.[Gln1201Pro]; [Gln1201Pro]	0.8	NA	≥1	NA	NA	NO	NO	NO	NO	NO	NO	Liver transplantation
48	NA	NA	c.[5741G > A];[1528C > T]; p.[Arg1914His];[Arg510*]	NA	NA	0	≥1	NA	Short stature; Delayed skeletal maturation; Skeletal dysplasia; Muscular hypotonia and hypotrophy	NA	YES	Motor delay; Intellectual disability	Hypogammaglobulinemia	NO	NO
49	NA	NA	c.[5740C > G];[6877delC]; p.[Arg1914Gly]; [Leu2293Cysfs*9]	0.5	NA	≥1	NA	NA	Short stature; Reduced bone mineral density; Delayed skeletal maturation; Skeletal dysplasia; Muscular hypotonia and hypotrophy	NA	YES	Motor delay; Optic atrophy	Hypogammaglobulinemia	YES	High pitched voice
50	NA	NA	c.[5761G > C]; [686dupT]; p.[Ala1921Pro]; [Ser230Glnfs*4]	0.4	NA	0	≥1	NA	NA	NA	NO	NO	Hypogammaglobulinemia	YES	NO
51	NA	NA	c.[6840G > T]; [6840G > T] p.[?];[?]	0.3	NA	0	≥1	NA	Short stature; Skeletal dysplasia; Delayed skeletal maturation	NA	NO	Optic atrophy; Motor delay	Hypogammaglobulinemia	YES	Rotary nystagmus; High pitched voice
52	NA	NA	c.[6966_6969delinsTC];[5547delC] p.[Gln2322Hisfs*18]; [Trp1850Glyfs*32]	4.5	NA	0	≥1	NA	NO	NA	NO	Optic atrophy; Motor delay	Hypogammaglobulinemia; Reduced NK cells	NO	Premature birth; Recurrent bacterial pneumonia
53	NA	NA	c.[767G > A];[2012 T > G]; p.[Cys256Tyr]; [Phe671Cys]	0.1	NA	0	≥1	NA	Short stature	NA	NA	Intellectual disability; Motor delay	NO	NO	NO
54	NA	NA	c.[2535G > T]; [5761G > C] p.[Trp845Cys]; [Ala1921Pro]	NA	NA	≥1	NA	NA	Short stature	NA	NA	NO	NA	NO	NO
55	M	Italy	c.[6840G > A];[686dupT] p.[Gly2238_Thr2280del];[Ser230fsGln*4]	NA	7	0	≥1	NA	Short stature; Skeletal dysplasia Reduced bone mineral density;	YES	NA	Neurodevelopmental delay with normal intelligence	Hypogammaglobulinemia	YES	Coeliac disease; Congenital bilateral glaucoma; Primary hypothyroidism
56	F	Italy	c.[6840G > A];[1501C > T] p.[Gly2238_Thr2280del];[Arg501*]	NA	4	0	≥1	NA	Short stature; Skeletal dysplasia	YES	NA	Neurodevelopmental delay with normal intelligence	Hypogammaglobulinemia	YES	Premature birth; Retinopathy of prematurity

*NA* Not available, *ALF* Acute liver failure, *LC* Liver crisis;

Patients 1 to 14 were published by Staufner C et al. [8]; Patients 15 to 16 were published by Segarra NG et al. [5]

Patient 17 was published by Hasosah MY et al. [11]; Patient 18 was published by Ono S et al. [7]

Patient 19 was published by Yucan Zheng [14]; Patient 20 was published by Xuerui Song [12]

Patient 21 was published by Jiali Gu [13]; Patient 22 was published by Wang J [15]

Patient 23 to 25 were published by Li JQ [9]; Patient 26 was published by Kortum [6]

Patient 27 to 28 were published by Regateiro FS [16] Patient 29–54 was published by Staufner [17]

Patient 55 to 56 were published by Carli [18]

Although the clinical manifestation of ILFS2 in this case report was typical, the definitive diagnosis of this disease depended on whole exome sequencing as it was important to rule out ALF caused by drugs, infection or simultaneous autoimmune abnormality. Many mutation sites in the NBAS gene have been reported [5, 7, 8]. Through NBAS sequencing, it was determined that three heterozygous mutations occurred in the patient under discussion. Specifically, *(c.3386C > T (p.Ser1129Phe)*, *c.1A > C (p.Met1Leu)* and *c.875G > A (p.Gly292Glu))*. Regarding the *c.3386C > T (p.Ser1129Phe)* mutation, Staufner et al. have reported that two patients who harbored this mutation similarly presented ALF without other extrahepatic manifestations [17]. The onset age of those two patients were 2.3 and 1.8 years respectively, close to the age of onset for our patient (2.9 years). Staufner et al. indicated that the *c.3386C > T (p.Ser1129Phe)* mutation affected the region coding for the Sec39 domain of the NBAS gene, which is necessary for tethering at the endoplasmic reticulum (ER) in the syntaxin18 [17]. The *c.1A > C (p.Met1Leu)* mutation is a likely pathogenic mutation (90% pathogenicity) according to the guidelines of the ACMG. Further, the software for the prediction of protein biological function have also indicated that this mutation is likely pathogenic. Thus, we speculated that the mutations including *c.3386C > T (p.Ser1129Phe)* and *c.1A > C (p.Met1Leu)* are associated with the IFS2 while the *c.875G > A (p.Gly292Glu)* mutation was likely nonpathogenic.

To date, the molecular pathogenesis of ALF in NBAS deficiency has remained unclear. The biological role of NBAS is two-fold. As a subunit of the syntaxin 18 complex which is thermally susceptible, it is associated with Golgi-to-ER retrograde transport [19]. On the other hand, NBAS plays an important role in nonsense-mediated mRNA decay control [20]. The dysfunction of the syntaxin 18 complex caused by NBAS deficiency may alter Golgi-to-ER retrograde vesicular trafficking, which in turn may lead to liver dysfunction and eventually, failure [8, 10].

Complete recovery of liver function in patients with IFLS2 can be achieved through comprehensive treatment. Therapeutic experience shows that early antipyretic therapy and treatment of the primary diseases that trigger fever is effective and able to prevent life-threatening ALF. Additionally, support treatments including intravenous infusion of lipid and glucose to maintain the internal environment stable and energy replete expedite recovery [8, 11]. Generally, liver transplantation is not considered unless intensive conservative treatment is ineffective. The patient in our study received antipyretics in addition to other conservative strategies including plasmapheresis (only in first episode), hepatoprotectives and intravenous glucose and electrolyte infusion. These treatments led to a remarkable recovery.

The present case detailed the course of a Chinese girl with ILFS2 presenting typical clinical characteristics of the disease-without the involvement of extrahepatic systems. The patient was found to harbor novel compound heterozygous mutations in the NBAS (*c.3386C > T (p.Ser1129Phe), c.1A > C (p.Met1Leu) and c.875G > A (p.Gly292Glu))*, two mutations of which have not been previously reported. Here we suggest that ILFS2 should be considered as a part of the differential diagnoses of children presenting with recurrent ALF triggered by fever. However, the definitive diagnoses of ILFS2 continues to depend on sequencing results of NBAS as well as the elimination of other potential causes of ALF including drug reactions, infections and autoimmune hepatitis. Early and effective antipyretic and support treatments such as intravenous lipid and glucose infusion are also recommended as these can prevent further liver crisis and lead to faster recovery.

## Data Availability

Not applicable.

## Abbreviations

ALF: Acute liver failure
NBAS: Neuroblastoma amplified sequence
ILFS2: Infantile liver failure syndrome-2
SOPH: Optic never atrophy and Pelger-Huët anomaly of granulocytes
Cre: Creatinine
CK: Creatine kinase
cTnI: Cardiac troponin I
WBC: White blood cell
CRP: C-reactive protein
ACMG: The American College of Medical Genetics Genomics and the Association for Molecular Pathology
ER: Endoplasmic reticulum

## References

[1] Narkewicz MR, Dell Olio D, Karpen SJ, Murray KF, Schwarz K, Yazigi N (2009). Pattern of diagnostic evaluation for the causes of pediatric acute liver failure: an opportunity for quality improvement. J Pediatr.

[2] Dhawan A (2008). Etiology and prognosis of acute liver failure in children. Liver Transpl.

[3] Vilarinho S, Choi M, Jain D, Malhotra A, Kulkarni S, Pashankar D (2014). Individual exome analysis in diagnosis and management of paediatric liver failure of indeterminate aetiology. J Hepatol.

[4] Maksimova N, Hara K, Nikolaeva I, Chun-Feng T, Usui T, Takagi M (2010). Neuroblastoma amplified sequence gene is associated with a novel short stature syndrome characterised by optic nerve atrophy and Pelger-Huet anomaly. J Med Genet.

[5] Segarra NG, Ballhausen D, Crawford H, Perreau M, Campos-Xavier B, van Spaendonck-Zwarts K (2015). NBAS mutations cause a multisystem disorder involving bone, connective tissue, liver, immune system, and retina. Am J Med Genet A.

[6] Kortum F, Marquardt I, Alawi M, Korenke GC, Spranger S, Meinecke P, et al. Acute liver failure meets SOPH syndrome: a case report on an intermediate phenotype. Pediatrics. 2017;139(1):e20160550. 10.1542/peds.2016-0550 28031453

[7] Ono S, Matsuda J, Watanabe E, Akaike H, Teranishi H, Miyata I (2019). Novel neuroblastoma amplified sequence (NBAS) mutations in a Japanese boy with fever-triggered recurrent acute liver failure. Hum Genome Var.

[8] Staufner C, Haack TB, Kopke MG, Straub BK, Kolker S, Thiel C (2016). Recurrent acute liver failure due to NBAS deficiency: phenotypic spectrum, disease mechanisms, and therapeutic concepts. J Inherit Metab Dis.

[9] Li JQ, Qiu YL, Gong JY, Dou LM, Lu Y, Knisely AS (2017). Novel NBAS mutations and fever-related recurrent acute liver failure in Chinese children: a retrospective study. BMC Gastroenterol.

[10] Haack TB, Staufner C, Kopke MG, Straub BK, Kolker S, Thiel C (2015). Biallelic mutations in NBAS cause recurrent acute liver failure with onset in infancy. Am J Hum Genet.

[11] Hasosah MY, Iskandarani AI, Shawli AI, Alsahafi AF, Sukkar GA, Qurashi MA (2017). Neuroblastoma amplified sequence gene mutation: a rare cause of recurrent liver failure in children. Saudi J Gastroenterol.

[12] Xuerui Song ZC, Cheng X, Zhou F, Zhou L, Zhao X, An Y (2019). Clinical and immunological characteristics of a case with ILFS2. Immunological J.

[13] Jiali Gu WW, Li LI, Zheng Y, Mao X (2019). A novel compound heterozygous mutation in NBAS gene causes SOPH syndrome and liver function damage. Chin J Pediatr.

[14] Yucan Zheng JL, Guo H (2018). One case of infantile liver failure syndrome-2. Chin J Pediatr.

[15] Wang J, Pu ZJ, Lu ZH (2018). Targeted next-generation sequencing reveals two novel mutations of NBAS in a patient with infantile liver failure syndrome-2. Mol Med Rep.

[16] Regateiro FS, Belkaya S, Neves N, Ferreira S, Silvestre P, Lemos S (2017). Recurrent elevated liver transaminases and acute liver failure in two siblings with novel bi-allelic mutations of NBAS. Eur J Med Genet.

[17] Staufner C, Peters B, Wagner M, Alameer S, Baric I, Broue P (2020). Defining clinical subgroups and genotype-phenotype correlations in NBAS-associated disease across 110 patients. Genet Med.

[18] Carli D, Giorgio E, Pantaleoni F, Bruselles A, Barresi S, Riberi E (2019). NBAS pathogenic variants: defining the associated clinical and facial phenotype and genotype-phenotype correlations. Hum Mutat.

[19] Aoki T, Ichimura S, Itoh A, Kuramoto M, Shinkawa T, Isobe T, et al. Identification of the neuroblastoma-amplified gene product as a component of the Syntaxin 18 complex implicated in Golgi-to-endoplasmic reticulum retrograde transport. Mol Biol Cell. 2009;20(11):2639–49. 10.1091/mbc.E08-11-1104 PMC2688544 19369418

[20] Longman D, Hug N, Keith M, Anastasaki C, Patton EE, Grimes G (2013). DHX34 and NBAS form part of an autoregulatory NMD circuit that regulates endogenous RNA targets in human cells, zebrafish and Caenorhabditis elegans. Nucleic Acids Res.

